# The Etiology of Acute Rheumatism

**Published:** 1893-11

**Authors:** 


THE ETIOLOGY OF ACUTE RHEUMATISM.—Sahli (*Deutches Archiv. fur klinische Medicin.*, Vol. LI, Parts 4 and 5)-reports a case of acute articular rheumatism which he observed during life and made a post-mortem and bacteriological examination upon. He concludes (1) That in a case of articular rheumatism not complicated by pus formation, he found a bacterium closely resembling the staphylococcus in the diseased joints, in the endocardial thickening, in the pericarditis, pleuritis, swollen bronchial glands and in the blood of the left heart. (2) It is probable that articular rheumatism is caused by a mild pathogenic bacterium. (3) The bacteriological researches make it possible that the so-called complications—endocarditis, pleuritis, etc., are really localizations of the disease and not true complications. He examined the blood and joint fluids of several cases of acute articular rheumatism and of erythema nodosum during life, and these examinations confirm him in the belief that these affections belong to the infectious diseases.—*Ex.*

